# Calcifying Pseudoneoplasm of the Neuraxis in the Posterior Fossa: A Case Report and Literature Review

**DOI:** 10.7759/cureus.21562

**Published:** 2022-01-24

**Authors:** Colin A Dallimore, Mica Quelle, Likowsky L Désir, Sunder Sham, Manju Harshan, Samuel J Wahl, Avraham Zlochower, Robert R Goodman, David J Langer, Randy S D'Amico

**Affiliations:** 1 Medicine, University of Kansas Medical Center, University of Kansas School of Medicine, Kansas City, USA; 2 Biology, Dwight School, New York, USA; 3 Medicine, City University of New York, New York, USA; 4 Pathology and Laboratory Medicine, Lenox Hill Hospital, Northwell Health, New York, USA; 5 Radiology, Lenox Hill Hospital, Donald and Barbara Zucker School of Medicine at Hofstra/Northwell, New York, USA; 6 Neurological Surgery, Lenox Hill Hospital, Donald and Barbara Zucker School of Medicine at Hofstra/Northwell, New York, USA

**Keywords:** rare, central nervous system, posterior fossa, capnon, calcifying pseudoneoplasm of the neuraxis

## Abstract

Calcifying pseudoneoplasm of the neuraxis are rare fibro-osseous lesions that can occur throughout the central nervous system. This paper reports one case of this lesion within the posterior fossa and contains a literature review of all cases documented within the posterior fossa to date. A 53-year-old female patient with a history of epiphora, facial irritation, and headaches was found to have a mass centered in the posterior fossa. The patient underwent surgical resection for removal of the mass. Upon review by pathology, the final diagnosis was consistent with calcifying pseudoneoplasm of the neuraxis.

## Introduction

Calcifying pseudoneoplasm of the neuraxis (CAPNON) are rare fibro-osseous lesions first described by Rhodes and Davis in 1978 [1]. Originally suspected to be neoplastic [1] or metastatic [2] in origin, recent data suggests that the granulomatous inflammation present in many CAPNONs reflects a reactive proliferative pathogenesis [3]. Although CAPNON lesions have been most commonly identified in the supratentorial regions of the CNS, they can occur throughout the central nervous system (CNS) with cases documented in the brain parenchyma, dura, and spine [3-10] Cranial lesions most commonly present with headaches and seizures [4]. Notably, radiographic diagnosis remains difficult as these rare lesions are often mistaken for other intracranial etiologies given their appearance on radiologic analyses. Thus, tissue biopsy is required for accurate diagnosis.

CAPNON affects patients between the ages of 30 and 64 years with a slight predilection for males. Little is otherwise known regarding this rare lesion. Surgical resection remains the current mainstay of treatment for symptomatic CAPNONs for diagnostic and therapeutic purposes. Here we present a case of a posterior fossa CAPNON treated with surgical resection and review the literature on this rare disease.

## Case presentation

**Figure 1:**
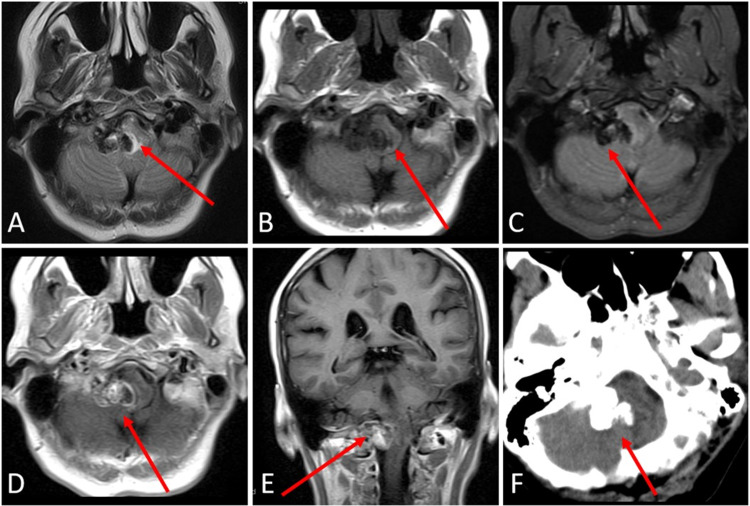
Preoperative imaging of a right posterior fossa calcifying pseudoneoplasm of the neuraxis (CAPNON). Axial T2 (A), T1 (B), and GRE (C)-weighted MRI of the brain demonstrates a predominantly T1 and T2 hypointense extra-axial mass centered in the posterior fossa on the right at the level of the lateral cerebellomedullary cistern. There is mass effect and T2 hyperintensity in the medulla compatible with vasogenic edema (arrow, A). There is blooming artifact on GRE (C) compatible with calcification. Axial (D) and coronal (E) post contrast T1-weighted images demonstrate heterogenous enhancement. Axial non-contrast CT head demonstrates a predominantly calcified mass centered in the posterior fossa in the right lateral cerebellomedullary cistern (F). GRE: gradient recalled echo

A 53-year-old female presented with a 4-year history of epiphora of the right eye, facial irritation, and remote ipsilateral brief headaches with a non-contributory past medical history. This irritation progressed to a persistent burning sensation along with episodes of shooting pain of the right face that were refractory to gabapentin. Persistence of the patient’s pain prompted an MRI brain that revealed a predominantly T1 and T2 hypointense extra-axial mass (Figures 1A-1E) and non-contrast CT head that demonstrated a predominately calcified mass (Figure 1F) centered in the posterior fossa on the right at the level of the lateral cerebellomedullary cistern.

Upon completion of imaging, management options were discussed, including a recommendation for surgical resection, but the patient opted for further monitoring of the lesion. The patient returned after two months with increased frequency and severity headaches and new-onset numbness in her right thumb and toes. Follow-up MR brain scan revealed no appreciable interval progression in size, enhancing characteristics or localized mass effect associated with the right posterior fossa enhancing mass. However, due to progression of symptoms, the patient ultimately decided to proceed with surgical intervention.

A far-right lateral approach was performed for suboccipital craniectomy and C1 laminectomy with electrophysiological monitoring of somatosensory and motor potentials of cranial nerves. The ipsilateral vertebral artery was identified and preserved. The dura was opened to expose the lateral cerebellum and then extended down to the level of C1 to expose the upper cervical spinal cord. A firm tumor outside the brainstem was encountered shortly after dissection in the cerebellopontine (CP) angle was initiated. A reduction in sensory potential response for the right arm was noted after successful removal of tumor tissue. No further manipulation was carried out in the brain stem region. The remainder of the extra-axial portion of the tumor, originating from the dura, was removed subsequently. Following the operation, the resected specimen was submitted to pathology for review.

**Figure 2:**
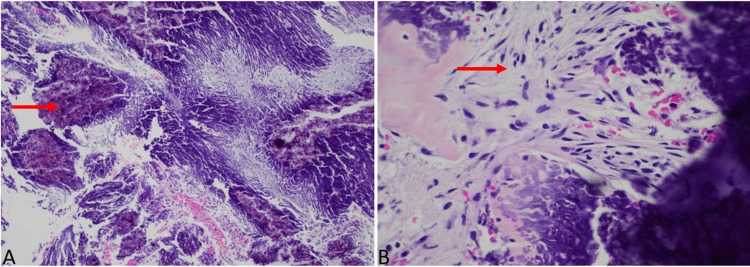
Histology of a right posterior fossa calcifying pseudoneoplasm of the neuraxis (CAPNON). Extensive calcifications with proliferation of spindle cells, H&E, 10× (A). Spindle cells, H&E, 40× (B). H&E: hematoxylin and eosin

Grossly, the specimen consisted of multiple fragments of tan-pink to white partially calcified tissue measuring 2.0 × 1.7 × 0.7 cm in aggregate. Histology examination showed extensive calcifications with a reactive proliferation of spindled mesenchymal cells (Figure 2), giant cell reaction, chondromyxoid matrix, ossification, and focal florid proliferation of spindle (piloid) astrocytes.

Differential diagnoses included meningioma, schwannoma, and glial neoplasm with calcifications. The spindle cells were negative for immune stains glial fibrillary acidic protein (GFAP) ruling out a glial neoplasm, epithelial membrane antigen (EMA), and progesterone receptor (PR) ruling out meningioma, and S100 excluding neural origin. The final diagnosis was consistent with CAPNON. Though CAPNONs have rarely been reported in association with low-grade gliomas and meningiomas, the astrocytic component, in this case, was interpreted as reactive as they did not label for BRAF V600 E and there was no intra-axial mass radiologically.

**Figure 3:**
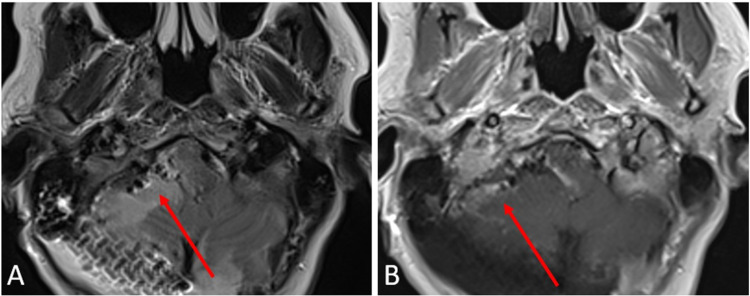
Postoperative imaging of a right posterior fossa calcifying pseudoneoplasm of the neuraxis (CAPNON). MRI - axial t2 (A) and axial t1 (B) status post-right suboccipital craniectomy with post-surgical changes in right cerebellar hemisphere. No evidence of residual posterior fossa mass. No evidence of residual enhancing mass.

In the immediate postoperative course, the patient was neurologically stable with persistent right-sided facial, arm, and leg numbness. Postoperative MRI showed no evidence of residual posterior fossa mass (Figure 3).

She developed right-sided neuropathic pain a week later and was started on Lyrica and advised to start Kessler Rehab. At six-month follow-up, the patient showed residual weakness, proprioceptive deficit, and numbness of the right arm and leg. Additionally, she experienced loss of left-sided temperature sensation. Patient reported continued guided physical therapy and the reintroduction of preoperative activities.

## Discussion

CAPNONs remain rare and mysterious entities despite an expanding body of literature characterizing their clinicopathologic features. CAPNON typically affects individuals with a median age of 47 years, although lesions have been identified in children as young as 2 years and adults as old as 83 years [4]. With little sex predilection, there is a slight male predominance (53.4%) [4]. Of the cases reported, 59.2% occur cranially and 40.8% occur in the spine. With cranial occurrence, 62% arise supratentorial with 33% occurring in the posterior fossa [4].

CAPNON presents with ambiguous symptoms mostly related to the location where it arises. The most common presenting symptoms with cranial CAPNON include headaches, neck pain, and/or seizures [3,4,11-19]. In cases limited to the spine, over half of patients report axial pain in the region of the lesion [4]. CAPNON in the posterior fossa often presents with cranial nerve defects due to the proximity of critical neurovascular structures [3,6,11-13,18,20-26]. Most commonly CN XI is affected, but these lesions affect a wide range of cranial nerves due to mass effect. These deficits can range from hoarseness and decreased hearing to gait disturbances and nerve paralysis, as outlined in Table 1. Because clinical presentations are nonspecific, it is important to understand the imaging and lab diagnostics that can help identify these lesions.

**Table 1:** CAPNON cases of the posterior fossa reported in the literature to date. CAPNON: calcifying pseudoneoplasms of the neuraxis; CN: cranial nerve; CP: cerebellopontine

Case	Source	Year	Age	Sex	Location	Presenting symptoms	Cranial nerve deficits	Initial treatment	Complications and adaptions	Progression	Follow-up (months)	Adjuvant treatment	Overall survival
1	Garen et al. [20]	1989	44	M	Right trigeminal ganglion	R stinging facial pain, in all three divisions of CN V	None	Gross-total resection	Mandibular division of the fifth nerve was sacrificed during surgery	No recurrence (symptoms completely relieved after surgery)	132	None	N/R
2	Bertoni et al. [3]	1990	31	M	Left cerebellopontine angle, jugular foramen, vertebral canal, oropharynx	Headache, hoarseness, jugular foramen syndrome	CN IX, X, and XI paralysis traversing the jugular foramen	Subtotal resection	None	Disease Recurrence 3 years after initial resection	156	Second debulking surgery 3 years after initial resection	13 years (cerebrovascular accident)
3			50	M	Foramen magnum	R neck and occipital area pain	None	Subtotal resection	None	No recurrence	42	None	N/R
4			48	M	Right cerebellar tonsil, spinal accessory nerve involvement	R XI nerve paralysis	R XI nerve paralysis	Gross-total resection	None	No recurrence	228	None	N/R
5			58	M	Jugular foramen	Hoarseness, decreased hearing	None	Intralesional excision	None	N/A	N/R	None	Lost to follow-up
6	Melville et al. [12]	1999	59	M	Foramen magnum	Neck pain, shuffling gait, decreased L hand sensation	None	Gross-total resection	None	No recurrence (stable after surgery with a stable gait disturbance)	24	None	N/R
7	Rodriguez et al. [21]	2008	67	F	Right cerebellar hemisphere (intra-axial)	Slightly brisk reflexes, (CAPNON was revealed incidentally)	None	Gross-total resection of the lesion and adjacent ependymoma	None	N/R	N/R	N/R	N/R
8	Ozdemir et al. [22]	2011	53	M	Foramen magnum	L facial pain, L leg monoparesis, gait disturbance	None	Gross-total resection	None	No recurrence	N/R	None	N/R
9	Hodges et al. [11]	2011	34	M	Left posterior fossa, left cilvus (mass effect on the brainstem and cerebellum)	Headache, tinnitus, fatigue, dizziness, blurry vision, L tongue deviation, tongue atrophy, L uvula deviation	CN XII paralysis (hypoglossal palsy)	Subtotal resection	A small portion of the lesion was adherent to the lower CN and the posterior inferior cerebellar artery, however, this portion was not resected to prevent further damage	No recurrence (stable with persistent hypoglossal palsy after surgery)	7	None	N/R
10	Muccio et al. [13]	2012	55	F	Right foramen magnum	Neck pain and hypoesthesia, hypoesthesia the right hemisoma	None	Gross-total resection	None	No recurrence (stable after surgery)	14	None	N/R
11	Kerr et al. [13]	2013	56	M	Right cerebellomedulary angle (mass effect on the brainstem)	Suboccipital headaches	None	Subtotal resection	A small portion of the lesion was adherent to the lower cranial nerves and the brainstem; however, this portion was not resected in order to prevent damage	Disease recurrence free (dysphagia and hoarseness after surgery)	6	None	N/R
12	Fatih et al. [15]	2014	59	F	Cerebellomedulary cistern	Headaches	None	Gross-total resection	None	N/R	6	N/R	N/R
13	Lu et al. [16]	2015	N/A	N/A	Right cerebellomedulary angle	Suboccipital headaches	None	Gross-total resection	None	N/R	N/R	N/R	N/R
14	Wiśniewski et al. [17]	2015	29	M	Right foramen magnum	Headaches, limited L rotation of the head	None	Gross-total resection	None	No recurrence (stable after surgery)	24	None	N/R
15	Ghaemi et al. [18]	2016	18	M	Interpenduncular cistern	Headaches, intermittent diplopia, limited downgaze, limited adduction of the eye, sluggish L pupillary response	Cranial nerve III palsy	Resection (Unspecified)	None	N/R (Experienced persisting diplopia after surgery)	N/R	N/R	N/R
16	Alshareef et al. [6]	2016	59	F	R cerebellomedulary angle, foramen magnum, intradural, extramedullary	Gait instability, balance difficulty, burning sensation of R face, itching sensation of R eye with watering, R deviation of palate	None	Subtotal resection	None	Disease recurrence free (stable after surgery, symptoms completely resolved 2 months after treatment)	12	None	N/R
17			72	F	Cerebellum	Tinnitus, ataxia	None	Gross-total resection	None	Disease recurrence free	N/R	None	N/R
18	Brasiliense et al. [19]	2017	67	F	Brain stem, frontal lobe	Recurrent focal seizures with impaired consciousness	None	Gross-total resection of the frontal lobe lesion (the brainstem lesion was not removed)	None	No recurrence and no evident change or growth in the brainstem lesion (seizure-free after surgery)	4	None	N/R
19	Nussbaum et al. [23]	2018	39	F	Right jugular foramen (in the region of the pars nervosa), right petrous apex, internal auditory canal	R hearing loss with intrusive right sides tinnitus	None	Gross-total resection	The labyrinth was resected in order to expose the lesion causing right-sided hearing loss	N/R (right-sided complete hearing loss after surgery)	N/R	N/R	N/R
20	Peker et al. [25]	2018	58	F	Left lateral cerebellomedullary junction	Recurrent dizziness	None	Total resection	None	Dizziness resolved 2 months post-surgery. No recurrence after 12 months follow-up	12	None	N/R
21	Yang et al. [24]	2020	57	M	Right cerebellopontine angle	Hoarseness, dysphagia, gait imbalance, gait ataxia, R cranial nerve IX, X, XI, and XII palsies	CN IX, X, XI, and XII palsy	Subtotal resection	Only the part of the lesion compressing the medulla was removed to avoid worsening cranial neuropathies	Slight disease progression, however, the patient remained clinically well overall (stable after surgery with persisting cranial neuropathies and improvement of ataxia)	15	None	N/R
22	Yan et al. [26]	2021	44	F	Right posterior CP skull base, clivus, jugular foramen	Occipital headaches, neck pain	None	Subtotal resection	None	Neck pain 2 months post-resection	12	None	N/R

When arising at the skull base, the radiographic differential associated with a CAPNON diagnosis has included chordoma, chondrosarcoma, vestibular schwannoma, and meningioma [3]. Despite this, a uniform T2 hypointensity without enhancement can help to rule out all but meningiomas [5]. This leaves calcified meningioma as the most common diagnosis that is still confused with CAPNON [5,6,18,20]. Absence of a dural tail can help separate CAPNON from calcified meningioma [24], but it can sometimes be difficult to definitively identify CAPNON from imaging alone.

Histopathologically, CAPNON displays distinctive histology that commonly feature: chondromyxoid regions, palisading spindle cells, fibrous stroma, calcifications, and psammoma bodies [4,12,24]. This helps differentiate these rare lesions from more common neoplasms on the differential.

Symptomatic CAPNON remain benign lesions and symptoms arise from mass effect on critical neurovascular structures. Evidence supports safe, maximal resection in symptomatic patients. These data can be extrapolated to posterior fossa CAPNON where published reports have pursued gross total or subtotal resection was performed to relive symptoms (Table 1). Twelve of the 22 identified cases listed had no disease recurrence at median follow-up of 14 months [3,6,11-14,17,19,20,22]. In seven of the 20 cases, recurrence was not reported, or patients were clinically improved with minimal recurrence over the duration of follow-up [3,15,16,18,21,23,24]. Only one of the reported cases had a recurrence at three years that required a second debulking surgery [3]. In all of the cases covered in this review, resection led to either stabilization of symptoms after surgery, or complete relief in some cases with only three recurrences over the average observation period of 37 months. No lesions required adjuvant treatment with chemotherapy or radiation. As a result, the efficacy of radiation therapy or radiosurgery to manage recurrences remains unknown. While data are limited by duration of published observations, safe maximal resection appears to be a safe and effective means of management of symptomatic disease. Resection however is not without complications. Postoperative deficits were identified in 20% of patients in posterior-fossa cases which likely reflect the proximity of symptomatic lesions to critical neurovascular structures and difficulties with resection of these calcified lesions. Given the benign nature of the disease and documented lack of recurrences following resection, preservation of neurologic function remains paramount to any surgical decision making.

## Conclusions

CAPNON is a rare nonneoplastic lesion that can manifest across the entire CNS. The paucity of information on CAPNON coupled with its radiographically nonspecific features makes diagnosis a challenge, often leading to misdiagnoses as other types of neoplastic lesions. As such, increasing awareness of this lesion can help avoid unnecessary and harmful management.
